# 1223. Utilizing Multiple Antimicrobial Stewardship Metrics to Trend Vancomycin Use in an Implementation Study

**DOI:** 10.1093/ofid/ofad500.1063

**Published:** 2023-11-27

**Authors:** Kaleb H Wolfe, Casey Smiley, Eileen Hasse, Nina Millman, Michael Chambers, Sage Hendrickson, Parmida Parvaz, Matthew Lokant, Nishant Patel, Rachael Pellegrino, Kimberly C Okafor, Christina Vojtek, Milner Staub, Christina T Fiske, Jamison Norwood

**Affiliations:** Vanderbilt University Medical Center, Nashville, Tennessee; Vanderbilt University Medical Center, Nashville, Tennessee; Vanderbilt University Medical Center, Nashville, Tennessee; Vanderbilt University Medical Center, Nashville, Tennessee; Vanderbilt University Medical Center, Nashville, Tennessee; Tennessee Valley Veterans Affairs Healthcare System, Nashville, Tennessee; Tennessee Valley Healthcare System, Nashville, Tennessee; Vanderbilt University Medical Center, Nashville, Tennessee; Vanderbilt University, Nashville, Tennessee; Vanderbilt University Medical Center, Nashville, Tennessee; VANDERBILT UNIVERSITY MEDICAL CENTER, Nashville, Tennessee; Vanderbilt University, Nashville, Tennessee; Vanderbilt University Medical Center, VA Tennessee Valley Healthcare System, Nashville, TN; Vanderbilt University Medical Center, Nashville, Tennessee; Vanderbilt University Medical Center, Nashville, Tennessee

## Abstract

**Background:**

Inpatient IV vancomycin use is often inappropriately prolonged. Methicillin-resistant *Staphylococcus aureus* (MRSA) polymerase chain reaction (PCR) nares swabs have shown high negative predictive values for many infections and can guide appropriate vancomycin de-escalation. Antibiotic stewardship programs (ASPs) often monitor vancomycin use in days of therapy (DoT) per 1000 patient days present (PDP). This measure can be affected, especially for smaller facilities, by changes in bed capacity and prolonged stays and thus may not accurately reflect vancomycin use. We evaluated whether alternative metrics of vancomycin use would show different impacts during a multi-step ASP intervention to reduce inappropriate vancomycin use at Veterans Affairs Tennessee Valley Healthcare System.

**Figure 1**

**Figure:**
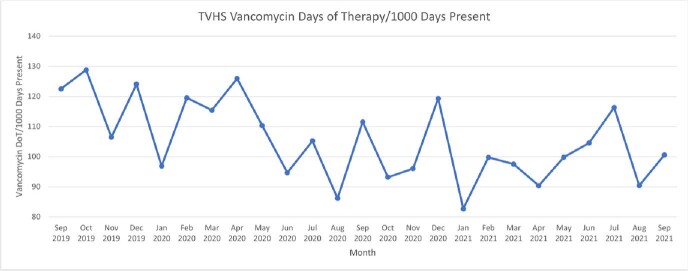

Run chart of vancomycin DoT/1000 PDP at the Tennessee Valley Healthcare System (TVHS) in two years prior to intervention.

**Methods:**

In September 2021, the ASP, with multiple stakeholders, initiated use of MRSA nasal swabs to inform vancomycin de-escalation. From 7/2022 to 3/2023, we performed iterative Plan-Do-Study-Act intervention cycles including educating medicine and surgical divisions on MRSA swab result interpretation, educating nurses on timely swab collection, replacing MRSA culture swabs with PCR swabs, and adding MRSA swab orders to vancomycin order sets. In April 2022, in addition to DoT/1000 PDP, we began measuring mean, median, and mode DoT per vancomycin course initiated by start week.

**Results:**

Despite multiple interventions beginning July 2022, vancomycin use in DoT/1000 PDP increased to a peak of 106 DoT/1000 PDP in September 2022. In that same period, median and mode DoT per vancomycin initiation remained steady at 2 days and 1 day, respectively.

**Figure 2**

**Figure:**
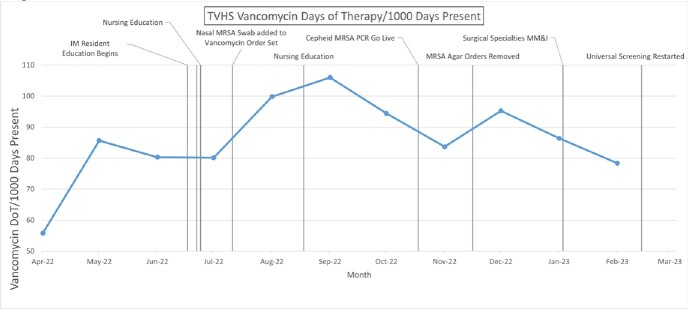

Run chart of vancomycin DoT/1000 PDP at the TVHS from April 2022 to March 2023. Specific ASP interventions are annotated on the dates they occur.

**Figure 3**

**Figure:**
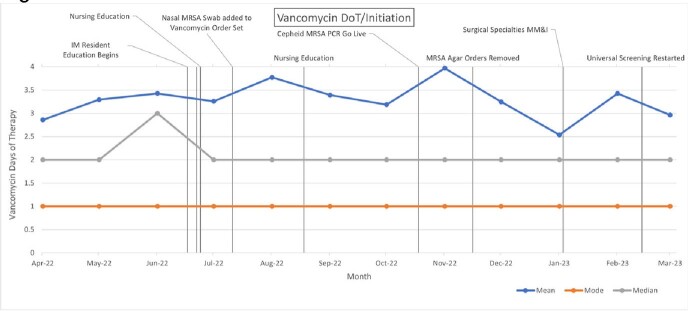

Run chart of mean, median, and mode days of vancomycin therapy per vancomycin course initiated at the TVHS from April 2022 to March 2023. Specific ASP interventions are annotated on the dates they occur.

**Conclusion:**

DoT/1000 PDP may not be the best metric to evaluate efforts to reduce inappropriate vancomycin use. By tracking mean, median, and mode of DoT per individual vancomycin course, we determined that most providers already practice rapid de-escalation, minimizing patient vancomycin exposure. This method also allows us to quickly detect changes in prescribing patterns and determine whether interventions were warranted. More evaluation of antibiotic use metric appropriateness based on desired outcome is needed in ASPs.

**Disclosures:**

**Milner Staub, MD, MPH**, Gilead: Stocks/Bonds|Johnson & Johnson: Stocks/Bonds

